# Use of conventional and alternative treatment strategies for a case of low back pain in a F/A-18 aviator

**DOI:** 10.1186/1746-1340-14-11

**Published:** 2006-07-04

**Authors:** Bart N Green, John Sims, Rachel Allen

**Affiliations:** 1 Contracted chiropractic physician, Naval Medical Center, San Diego, Marine Corps Air Station Miramar Branch Medical Clinic, Bldg 2496 Bauer Rd, San Diego, CA, USA; 2 Flight Surgeon, VMFAT-101, Marine Corps Air Station Miramar, San Diego, CA, USA; 3 Physical Therapist, Saint Michael's Hospital, Steven's Point, WI, USA

## Abstract

**Background:**

Low back pain can diminish jet pilot concentration and function during flight and be severe enough to ground pilots or cause decreased flying time. The objective of this case report is to present an example of the integration of chiropractic care with conventional treatments for the management of low back pain in a F/A-18 aviator.

**Case presentation:**

The patient had insidious severe low back pain without radiation or neurological deficit, resulting in 24 hours of hospitalization. Spinal degeneration was discovered upon imaging. Four months later, it still took up to 10 minutes for him to get out of bed and several minutes to exit the jet due to stiffness and pain. He had discontinued his regular Marine Corps fitness training due to pain avoidance. Pain severity ranged from 1.5–7.1 cm on a visual analog scale. His Roland Morris Disability Questionnaire score was 5 out of 24. The pilot's pain was managed with the coordinated efforts of the flight surgeon, physiatrist, physical therapist, and doctor of chiropractic. Following this regimen he had no pain and no functional disability; he was able to fly multiple training missions per week and exercise to Marine Corps standards.

**Conclusion:**

A course of care integrating flight medicine, chiropractic, physical therapy, and physiatry appeared to alleviate pain and restore function to this F/A-18 aviator with low back pain.

## Background

Low back pain (LBP) is a common problem associated with significant losses in work time in the general population [1]. While LBP has been studied extensively in the literature for many populations, few clinical studies discuss LBP in fighter jet aviators. Neck pain in fighter pilots receives much attention, yet spinal disorders leading to back pain are reported to be 2 times more common in fighter aviators than other pilots [2]. One survey reports that fighter pilots have a significantly greater prevalence of chronic LBP, pain requiring bed rest and pain radiating into the leg compared to fixed wing transport and helicopter pilots [3]. Spinal pain can be serious for high performance aviators and severe enough to ground pilots or cause decreased flying time (17% for fighter pilots) [4]. Spinal disorders and LBP are reported to be exacerbated by flight, result in disability [5] and in non-waiver of flight disqualification in approximately 25% of US Navy and US Marine Corps (USMC) aviators applying for it [6]. Back pain diminishes pilot concentration and function during flight [2]. Drew [4] reports that spinal pain significantly limits flying performance for 20% of the fighter pilots he studied.

Investigators are curious about aviators' use of alternative treatments to medicine to manage LBP, however little research is reported in this area. Three studies mention alternative management strategies and chiropractic care is mentioned briefly in 2 of these. Simon-Arndt et al [2] state that there is anecdotal evidence that pilots visit doctors of chiropractic. Drew [4] specifically queried aviators on their use of chiropractic care and found that doctors of chiropractic were in fact used by some pilots for spinal pain management. Although chiropractic services have been integrated into several US military treatment facilities since 1995 [7], the role of chiropractic care and how it is integrated with other health care services for military aviators is unreported. This case describes an example of such integration provided at a military treatment facility to manage LBP in a fighter pilot.

## Case presentation

A 36-year-old male USMC F/A-18 aviator instructor with 15 years of flying experience had a severe episode of acute LBP without radiation or neurological deficit. The patient did not recall any specific traumatic incident that initiated the pain, but he did have a history of multiple LBP events, some of which included radiation into the legs. When the LBP began he immediately consulted the squadron flight surgeon and was prescribed naprosyn, diazepam and hydrocodone/acetaminofen for pain control, confined to quarters to rest and imaging was obtained. The pain worsened, resulting in hospitalization later that day. He was observed for 24 hours and given a methylprednisone dosepack. Upon discharge from the hospital, he was confined to quarters for 72 hours and not allowed to return to flying until cleared by the flight surgeon. The flight surgeon cleared the patient to fly and ordered consults to neurosurgery and physical therapy for further evaluation and treatment.

**Figure 1:**
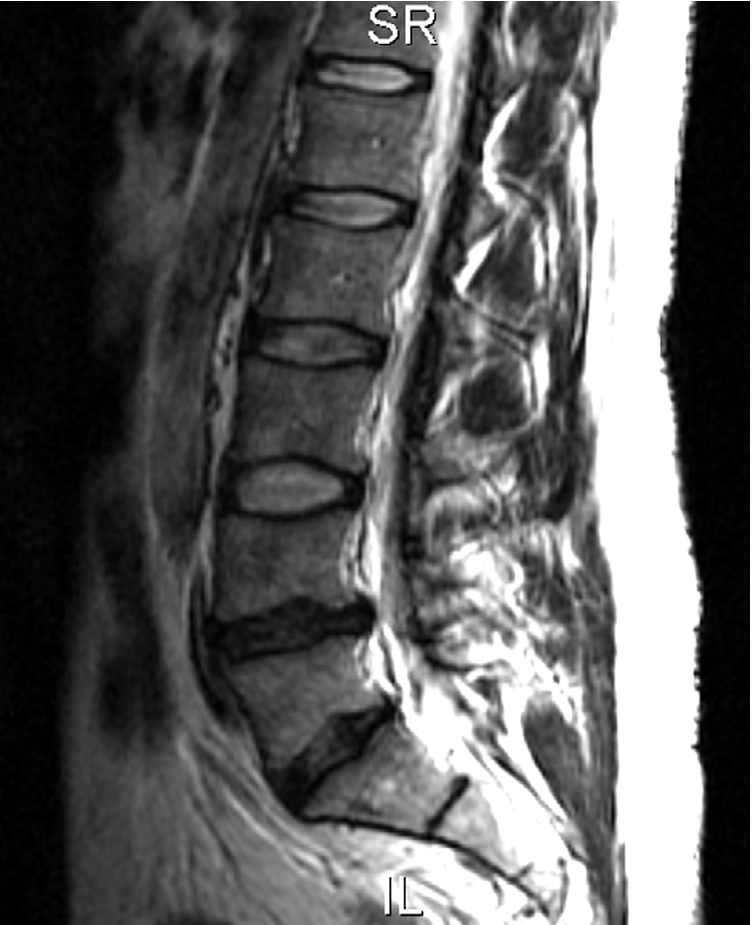
T2 weighted sagittal MRI demonstrating a loss of normal height and signal involving the L4–L5 disc and a broad-based paracentral disc bulge that contacted the thecal sac (arrow). L5-S1 disc degeneration was also present.

Plain film radiographs showed mild narrowing of the L4–L5 intervertebral disc space and mild sclerotic changes of the posterior elements of L5. MRI demonstrated a loss of normal height and signal involving the L4–L5 disc and a broad-based left paracentral disc bulge contacting the thecal sac and causing mild narrowing of the central spinal canal at the L4–L5 level with mild to moderate left neural foraminal narrowing and L5 nerve root contact (Fig 1). A lesser degree of L5-S1 disc degeneration was present with a broad-based disc bulge causing no central canal or neural foraminal narrowing.

The flight surgeon coordinated the ordering and follow up of the patient's various clinical consults. The flight surgeon instructed the patient to take the anti-inflammatory medication as needed and gradually returned him to non-impact exercise, including walking and working out on an elliptical machine to pain tolerance.

Following neurosurgical evaluation the patient was provided concurrent consultations with the hospital physiatrist and physical therapist. The physiatrist provided osteopathic manipulation and home exercises, including prolonged prone lumbar extension and hip adductor stretching, which provided some pain diminishment. Acupuncture was attempted once, wherein he reported an increased sensation of lumbar muscle spasm. Acupuncture was discontinued.

The physical therapy regimen was begun 2 weeks after hospitalization and included McKenzie exercises (standing and prone repetitive standing lumbar extension), stretching (hamstrings, single knee-to-chest, quadriceps, gluteals, hip flexors) and an educational self-treatment booklet. The patient experienced some relief with the McKenzie exercises, hamstring stretching and self mobilization/stretching of the gluteal muscles and spine by drawing his leg over to the opposite side of his trunk. His pain and range of motion improved approximately 50%. A course of moist heat packs and mechanical lumbar traction (15 min, supine position with hips and knees flexed 90 degrees, intermittent pull for 15 s, starting at 10 lbs greater than 50% body weight and increasing 10 lbs each treatment) was attempted to further his progress. He continued doing the prescribed exercises and noted some improvement but pain elimination was refractory. At this time, the physical therapist discussed the case with the chiropractor, who is located in the same clinic at the air station, and subsequently ordered a consultation for chiropractic.

During the chiropractic consultation, the patient's pain was located in the lumbar region bilaterally, described as "intense spasm", and without any radiation or symptoms of neurological involvement. His pain was consistently worse in the morning; he reported that it would take up to 10 minutes for him to get out of bed due to stiffness and pain. He was concerned because this episode of LBP was of longer duration (4 months) than any prior episodes. He had discontinued his regular Marine Corps fitness training (running, sit-ups, pull-ups) because of pain exacerbation. He experienced increased pain after basic fighter maneuver flights, reporting that it would take as long as 15 minutes to get out of the jet and climb to the ground after flying. He stated that even though he was on flying status he would sometimes ask to be removed from the flight schedule because his back hurt too much to fly; on these days he would stay at work and perform other tasks. He rated the severity of pain at 1.5 cm on a 10 cm visual analog scale [8], 7.1 cm upon waking and (in retrospect) 9.5 cm on the visual analog scale as the pain experienced during hospitalization. His modified Roland Morris Disability Questionnaire [9] score was 5 out of 24 functional disability indicators. Lumbar spine active ranges of motion were full with pain at the end range of flexion and extension and when returning to a neutral posture from these end ranges. Hamstring length was approximately 60 degrees bilaterally and the gluteus medius had approximately 50% of its normal passive length. Trigger points were palpated in the right gluteus medius and bilateral lumbar paravertebral and quadratus lumborum muscles. There were no indicators of lumbosacral nerve root or cord compression (ie, negative Valsalva, Kemp's test, straight leg raise). Lower extremity sensation, motor and deep tendon reflex testing were normal. Given his age, nature of the clinical findings, and the imaging results, it was assumed that he was experiencing phase II spinal degeneration (clinical instability) as described by Kirkaldy-Willis [10].

The chiropractor and flight surgeon discussed the case to insure that the flight surgeon was informed of the course of care. The chiropractor and the physical therapist discussed the case to insure that the care that the patient was receiving was complimentary and that any duplicate home exercises were planned redundancy. Thus, at this point in time, the patient was receiving care from the flight surgeon, physical therapist, and chiropractor and had 1 follow up visit scheduled with the physiatrist.

The 3 goals of chiropractic care were: 1) pain control; 2) ability to continue flight duties and USMC fitness training; 3) maintenance of aerobic fitness. Table 1 summarizes the chiropractic treatment interventions and outcome measures at periodic reassessments. Details regarding treatment are presented below. Directions of force for chiropractic manipulation were selected by identifying areas of tenderness, asymmetry, restricted planes of active and passive range of motion, motion palpation, tight musculature and other indicators as described by Bergman and colleagues [11]. Chiropractic manipulation of the thoracolumbar junction, L5-S1 level and the sacroiliac joint typically involved a side-posture high-velocity, low-amplitude short lever maneuver [11]. Grade IV mobilization [11] was used on several occasions when joint endfeel was not extremely stiff or if the patient was unable to tolerate a high velocity force. Active myofascial release treatment and ischemic compression as described by Barnes and Leahy were used to treat tight muscles and trigger points [12,13]. These muscles and surrounding joints were also stretched using the proprioceptive neurological facilitation maneuvers of post-contraction stretch and post-isometric relaxation [14]. The patient was instructed to self-administer trigger point ischemic compression to the gluteus medius by lying in the lateral recumbent position on top of a tennis ball and to stretch the muscle immediately afterward. He was instructed to continue the helpful stretches provided by the physiatrist and physical therapist, to stretch his low back before flying and to see his flight surgeon if his pain worsened.

**Table 1:** Outcome measures and treatment strategies during chiropractic care.

**Tx#/Wk#**	**Functional Outcomes**	**Pain Control**	**Flight/USMC Fitness Training**
1/1	• VAS = 1.5 current, 7.1 upon waking, 9.5 worst •Medication needed for pain control • RMDQ = 5/24 • Significant pain with flight • Unable to run/do sit-ups	• HVLAM [11] (T11-L2 & SI joint) • AMRT [12,13] & PIR or PCS [14] of (g. medius, g. max, QL and PVTs) • Home TrP Tx (g. medius) • Moist heat pack • See flight surgeon if pain increased	• G. medius stretch (2 @ 30 sec) • Double knee-chest stretches (10 reps @ 10 sec each) • Continue stretches recommended by PT and DO • Walking, elliptical and bicycle to tolerance

5/5	• No sharp pain • No medication needed to control pain • Mild ache after flying high G several times per week • Minimal pain with activities of daily living	Same as above	As above + • Supine leg raises (Dead Bugs 50 reps) • Static crunch to tolerance (90 sec) • Prone isometric core endurance (plank) for 90 sec

11/15	• Verbal pain scale = 0 • Mild tightness associated with prolonged sitting • Full activities • Able to perform plank exercise > 2 minutes • No pain with running	Periodic HVLAM, PCS and AMRT as necessary	As above + • Oblique crunches (50/side) • Isometric side bridge (60 sec) • Static lunge psoas stretch • Oblique crunches on gym ball • Latissimus pull downs • Seated rows • Gradual return to running

16/30	• VAS = 0 • RMDQ = 0/24 • Able to perform plank exercise > 120 sec, side-bridges > 60 sec, 100 crunches in < 120 sec, 50 oblique crunches per side • Able to fly multiple times/wk including long and high G flight with only mild tightness afterward • Passed required physical fitness test with no pain	No treatment required	• Maintain core stability and coordination exercises as part of routine exercise • 3 mi run 3x/wk, elliptical or stationary bike on other days

Tx = treatment; Wk = week; VAS = visual analog scale; RMDQ = Roland Morris disability questionnaire; HVLAM = high velocity low amplitude manipulation; T = thoracic; L = lumbar; AMRT = active myofascial release technique; PIR = post-isometric relaxation; PCS = post-contraction stretch; QL = quadratus lumborum; PVT = paravertebral muscles; TrP Tx = trigger point therapy

By the 5^th ^chiropractic treatment the patient reported there were no episodes of sharp "muscle spasm" pain in the previous week but periodic stiffness was experienced upon waking in the morning or after long periods of time in the jet. He was on regular flying status and he had discontinued taking any medication. The patient reported that the physiatrist had provided him with a home TENS unit for pain control, which provided relief at the end of long days in the jet or after prolonged sitting. At this point in time he was released from care by both the physiatrist and physical therapist and instructed to continue his home exercises and to return for care if symptoms worsened. Functional spinal stability was assessed by the chiropractor at the fifth office visit and the patient exhibited difficulty stabilizing his spine when asked to perform simple non-weight bearing movements called dead bug exercises [15]. He had no pain while performing a static crunch core endurance exercise. Treatment was modified to include core stabilizing exercises (Table 1).

Subsequent chiropractic office visits focused on furthering the patient's torso function and insuring coordinated care between the flight surgeon and the chiropractor. These office visits were supplemented with some form of manipulation/mobilization as deemed necessary. Therapeutic exercises were made more difficult and targeted strength, endurance and proprioception of the lumbar extensors, oblique abdominal muscles and other torso stabilizers [15]. Tight psoas muscles were also addressed with home-based stretching (Table 1). Summarily, the patient had 15 chiropractic office visits where he received care over a 26 week period. Office visits progressed from passive pain control techniques to active functional rehabilitation procedures and included the following treatments (frequency): high-velocity, low-amplitude manipulation (10); grade IV mobilization (8); proprioceptive neurological facilitation (6); myofascial release/ischemic compression (14); therapeutic exercise (7); moist heat pack application (2).

At a follow-up visit 1 month after his last treatment he was pain free and had full function. He was flying multiple training missions per week including high G flights and sorties of several hours in duration and had passed his required physical fitness test (100 sit-ups in 2 minutes, 3 mile run in less than 29 minutes and a minimum of 3 pull-ups) the week prior with no pain. He felt that the only provocative factor for minimal LBP at that time was flying the jet. The physical examination was normal; he was released from care and encouraged to maintain his core stabilization and overall fitness program.

## Discussion

The physical demands of the F/A-18 aviator are extreme. In addition to the physical requirements necessary to pilot the jet, US Marines must also maintain a high degree of physical fitness that is tested twice a year in a physical fitness test. Therefore, the management plan for this patient was directed at restoring his work capacity both in the jet and on the ground without having him restricted from flight by a light or limited duty status. Strategies to develop fitness of the lumbar region of aviators have been suggested, including postural, stretching exercises and core stability exercises [2]. Yet, no reports were found in the peer-reviewed literature to describe the content or effectiveness of such programs for jet aviators. Drew [4] reported that 54 of 79 high performance pilots used some form of stretching or exercise to prevent spinal symptoms. However, it is unknown how these pilots derived such programs (ie, self-taught vs. provided by health care provider) or if the exercises performed were actually appropriate for the spine. This case illustrates the rationale and types of prescribed therapeutic exercise for an aviator with LBP. Further research to investigate the use of lumbar exercises for pilots is necessary.

G forces are commonly cited as a cause of back pain in high performance aircraft pilots [2,16]. However, there is controversy. Voge et al [5] found no significant differences between aircrew and non-aircrew individuals until 1985, when the rates for aircrew fell below those of non-rated officers. They concluded that moderate G exposure did not seem to be a predictor of back disability. Summarily, there is no confluence of high quality evidence about this topic. Simon-Arndt et al [2] postulate that problems involving the back involve many microtraumas incurred during flight. They state that the G forces affect the pilot by compressing and jolting the spinal column and that the effects of G forces have been linked to lesions in the ligaments around the vertebrae and to the manifestation of latent thoracic and lumbar arthritis [2]. The present case shows a degenerative spine, but it is unknown if flying the F/A-18 was the cause.

Time and resources allocated to training and maintaining fighter aviators are extensive. For these reasons, as well as peer-pressure, self-esteem and pay, pilots are reluctant to disclose back pain for fear of being grounded [2]. Flight surgeons are designated first points of contact for pilots and it has been found that when high performance aviators do relent to seeking medical care for spinal problems, the flight surgeon is usually the first person they see [4]. Flight surgeons are trained extensively in the nuances of aviation medicine and the numerous regulations pertaining to aviation. Most flight surgeons are accustomed to collaborating with physical therapists and physiatrists but not necessarily doctors of chiropractic. Chiropractors are trained extensively in musculoskeletal pain management and managing non-surgical spinal conditions without the use of pharmaceutical agents [17]. Thus, it seems that these providers can serve as valuable allies to aviators experiencing spinal problems as long as there is clear communication between the various providers during patient management. It has been the experience of the authors that such communication is easily maintained in a branch medical clinic environment.

The natural history of LBP must be considered as a plausible explanation for this patient's recovery. There is relatively little quality information available on the natural history of LBP [18]. Patients usually experience rapid improvement in the first 3 months after LBP has occurred. However, of those patients initially off work because of LBP, 16% remain off work 6 months later and 62% still have pain at 12 months. Recurrences of pain and work absence are common in the year following the onset of LBP [18,19]. Comparing the patient in this case to what is known of the natural history of LBP, his initial improvement followed the trend for patients to experience rapid improvement in the first 3 months, and he did experience recurrences of pain in the ensuing 12 months. However, he was able to return to work quickly, even if it meant doing non-flying tasks, and continued to demonstrate improvements after the third month with LBP while he continued to fly and subject the spine to peak forces. It is conceivable that a multitude of variables, or combination of them, influenced his improvement, including the following: chance, chiropractic intervention, multidisciplinary management, natural remission, dose-response effect, placebo effect. As a retrospective case report, this case does not attempt to control for all variables. Its purpose is merely to describe and discuss a previously unreported intervention for pilots; the case suggests that a traditional course of care augmented with chiropractic treatment available at Department of Defense military treatment facilities may be of benefit to US fighter aviators with LBP. It is recognized that some treatment methods presented in this case report are not novel and other providers care for aviators using similar methods or practice models, but to date none have been reported in MEDLINE. It is hoped that this paper will stimulate further discussion on this topic.

## Conclusion

The addition of chiropractic care to the multidisciplinary management of this F/A-18 aviator with chronic LBP appeared to help alleviate pain and restore function. An appropriately powered prospective study would help determine the value of this type of treatment approach in this unique population.

## Competing interests

The first author is a contracted health care provider to the US Navy; there are no other competing interests to declare.

## Authors' contributions

BNG conducted the literature review and drafted the manuscript. JS and RA assisted in drafting the manuscript. All authors read and approved the final manuscript.
